# Automated quantification of the Alberta Stroke Programme Early CT Score (ASPECTS) and infarct core on diffusion-weighted imaging in acute ischaemic stroke: multi-centre validation and severity stratification

**DOI:** 10.1093/braincomms/fcag053

**Published:** 2026-02-24

**Authors:** Lai Wei, Ting Xiao, Hao Wang, Lei Shi, Qian Xi, Ming Liu, Huali Xu, Kangwei Zhang, Xiang Zhou, Yu Luo, Peijun Wang

**Affiliations:** Department of Radiology, Shanghai Fourth People's Hospital, School of Medicine, Tongji University, Shanghai 200434, China; School of Information Science and Engineering, East China University of Science and Technology, Shanghai 200235, China; School of Information Science and Engineering, East China University of Science and Technology, Shanghai 200235, China; School of Information Science and Engineering, East China University of Science and Technology, Shanghai 200235, China; Department of Radiology, Shanghai East Hospital, Tongji University School of Medicine, Shanghai 200120, China; Department of Radiology, Xinhua Hospital Affiliated to Shanghai Jiaotong University School of Medicine, Shanghai 200092, China; Department of Radiology, Putuo Hospital, Shanghai University of Traditional Chinese Medicine, Shanghai 200062, China; Department of Medical Imaging, Tongji Hospital, School of Medicine, Tongji University, Shanghai 200065, China; Institute of Medical Imaging Artificial Intelligence, Tongji University School of Medicine, Shanghai 200065, China; Department of Medical Imaging, Tongji Hospital, School of Medicine, Tongji University, Shanghai 200065, China; Institute of Medical Imaging Artificial Intelligence, Tongji University School of Medicine, Shanghai 200065, China; Department of Radiology, Shanghai Fourth People's Hospital, School of Medicine, Tongji University, Shanghai 200434, China; Department of Medical Imaging, Tongji Hospital, School of Medicine, Tongji University, Shanghai 200065, China; Institute of Medical Imaging Artificial Intelligence, Tongji University School of Medicine, Shanghai 200065, China

**Keywords:** acute ischaemic stroke (AIS), diffusion-weighted imaging (DWI), the Alberta Stroke Programme Early CT Score (ASPECTS), corevolume, deep learning (DL)

## Abstract

The purpose of this study was to develop and validate a deep learning framework for simultaneous automated quantification of diffusion-weighted imaging-the Alberta Stroke Programme early CT score (DWI-ASPECTS) and infarct core volume in middle cerebral artery acute ischaemic stroke (MCA-AIS) and to evaluate its clinical utility for severity stratification in multi-centre settings. A cohort of 738 patients diagnosed with MCA-AIS from four centres was divided into a train set (*n* = 408), a validation set (*n* = 116), an internal test set (*n* = 60) and an external test set (*n* = 154). A 3D U-Net architecture was trained for simultaneous infarct segmentation and the ASPECTS region analysis. Model performance was compared against expert neuroradiologists using intraclass correlation coefficients and the Spearman correlation coefficient. Furthermore, we investigated the correlation between ASPECTS deduction frequency, ASPECTS score, core volume and the severity of MCA-AIS. The 3D U-Net model showed a high correlation with manual segmentation, achieving a Dice coefficient of 0.801 and a Spearman correlation coefficient of 0.988 for volume measurements in the external test set. In both the internal and external test sets, the automated ASPECTS by the deep learning (DL) model (Automated), manual ASPECTS by raters (Raters) and manual ASPECTS by raters on DWI images registered with the template (Raters_template) exhibited a strong correlation and excellent agreement. The cortical regions (M1–M6) were particularly relevant in patients classified into the moderate–severe group. The threshold values for the mild group and moderate–severe group on receiver operating characteristic curve analysis for DWI-ASPECTS and Core volume, were 6 and 27.86 mL, respectively. The DL model demonstrated comparable performance to neuroradiologists’ evaluation, potentially serving as an ancillary tool for physicians in making urgent clinical decisions. The severity of MCA-AIS was significantly associated with the specific ASPETCS regions and core volume, which may aid in identifying moderate–severe MCA-AIS.

## Teaser

Wei *et al.* report that a novel deep learning model fully automates the Alberta Stroke Program Early CT Score (ASPECTS) scoring and infarct core volume measurement from diffuse-weighted imaging (DWI) scans. This tool was validated on multi-centre data and may aid in rapid stroke assessment.

## Graphical Abstract

**Graphical Abstract:**
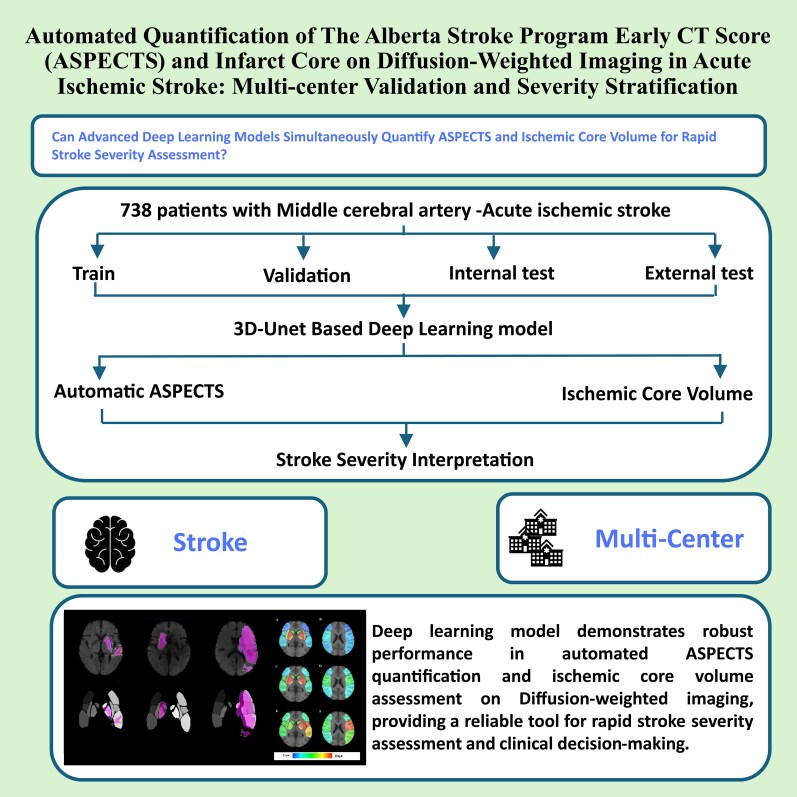

## Introduction

The Alberta Stroke Programme Early CT Score (ASPECTS) and ischaemic core volume (Core) represent complementary imaging biomarkers for assessing acute ischaemic stroke (AIS) severity. ASPECTS is a 10-point semi-quantitative scoring system for assessing the degree of early ischaemic changes in the middle cerebral artery–acute ischaemic stroke (MCA-AIS). ASPECTS has been widely utilized to identify patients who are presumed to have high risks for intracerebral haemorrhage and poor clinical outcomes.^1,2^ The AHA/ASA (American Heart Association/American Stroke Association) guidelines recommended ASPECTS ≥ 6 for selecting patients eligible for endovascular treatment (EVT).^3,4^ Estimating ischaemic core volume usually requires computed tomography perfusion (CTP) or diffusion-weighted imaging (DWI). The core volume derived from CTP or DWI was also utilized to assess the eligibility for EVT in a subset of patients in several authoritative trials.^5,6^

As is well-established, both ischaemic core volume calculation and ASPECTS scoring are experience-dependent and time-consuming, leading to inter-rater variability. These limitations underscore the need for automated, objective and reproducible methods for Core and ASPECTS assessment.^7,8^ Recently, deep learning (DL) models offered a potential solution to this problem.^9-11^ Several studies have reported the excellent performance of the automatic algorithm on the ischaemic core segmentation and volume calculations using DWI images,^12-15^ while other investigations have explored the application of ASPECTS scoring software or DL-based approaches for the binary classification of ASPECTS on CT images. However, existing research remains predominantly constrained by single-centre study designs and limited sample sizes. Importantly, no prior study has successfully integrated the automated assessment of both ASPECTS and ischaemic core volume into a unified, clinically applicable framework.

DWI represents the most sensitive imaging modality for detecting acute ischaemic stroke, offering superior tissue contrast compared to CT. DWI-ASPECTS, derived from CT-ASPECTS, enables more precise assessment of infarct core regions. Building upon these advancements, this study aimed to develop and validate a novel DL model capable of providing integrated ‘one-stop’ automated ASPECTS scoring and infarct volume quantification based on DWI images from four independent centres. We rigorously evaluated the performance of the DL model in comparison to the evaluation and segmentation of neuroradiologists through standardized training, validation and internal and external testing processes. Furthermore, we employed the DWI-ASPECTS and infarction volumes generated by the DL model to preliminarily interpret the severity of MCA-AIS through both anatomical regional involvement and volumetric quantification, thereby providing initial insights into the clinical application of our DL model.

## Materials and methods

**Figure 1:**
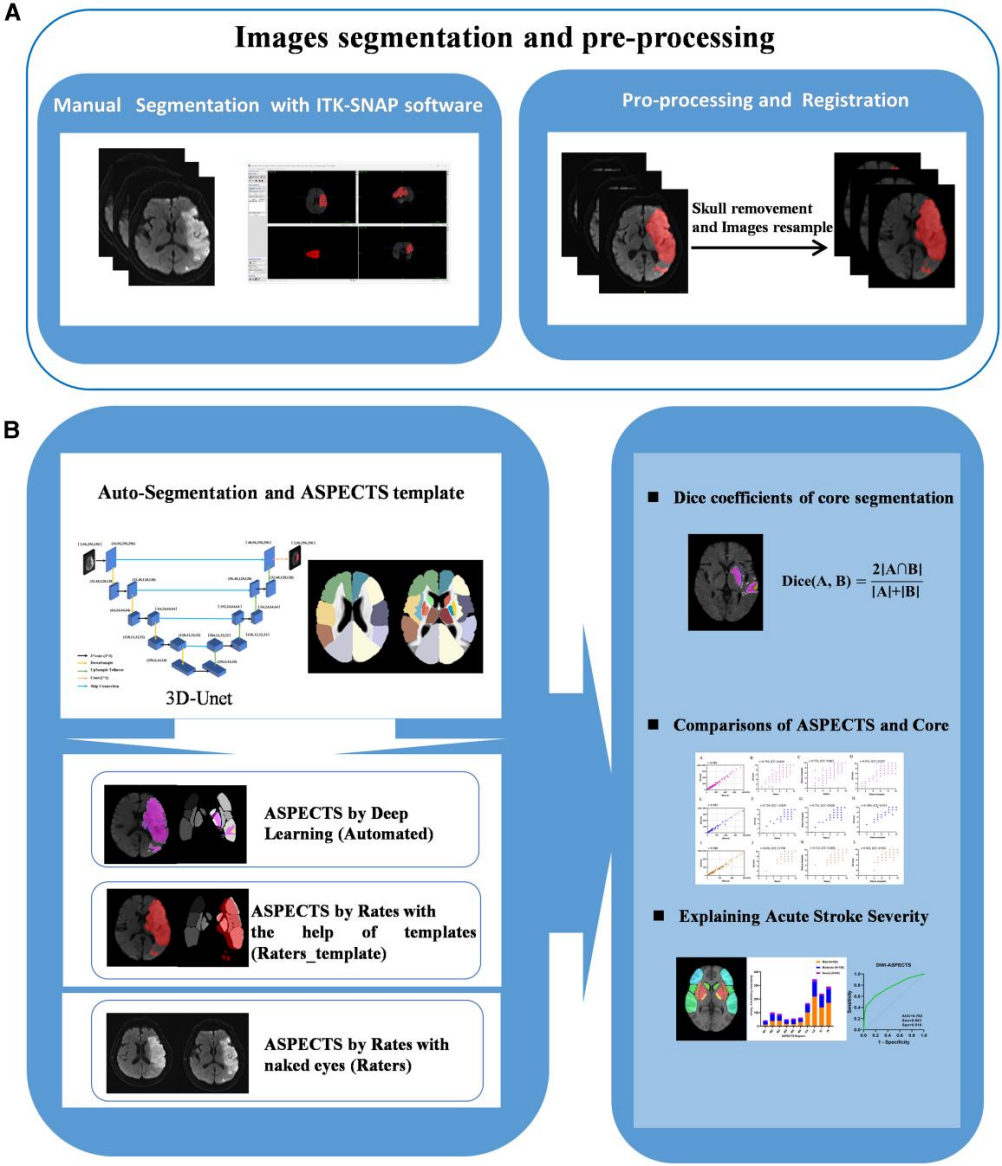
**The process framework diagram in this study.** (**A**) Image segmentation and pre-processing. (**B**) Model construction and clinical interpretation. This panel illustrates the construction and evaluation of the automated core volume calculation and ASPECTS scoring model, and the interpretation of stroke severity utilizing outputs by the automated DL model. ASPECTS = the Alberta Stroke Programme Early CT Score, 3D-Unet = Three-Dimensional U-Net.

This study constructed a deep learning architecture using Magnetic Resonance Imaging (MRI)-DWI data from four centres, enabling the automated quantification of infarct core volume and ASPECTS scoring for AIS. The model’s output was utilized to elucidate the severity of AIS. Figure 1 provides a structured and comprehensive framework of this study, encompassing the following steps.

### Study population

**Figure 2:**
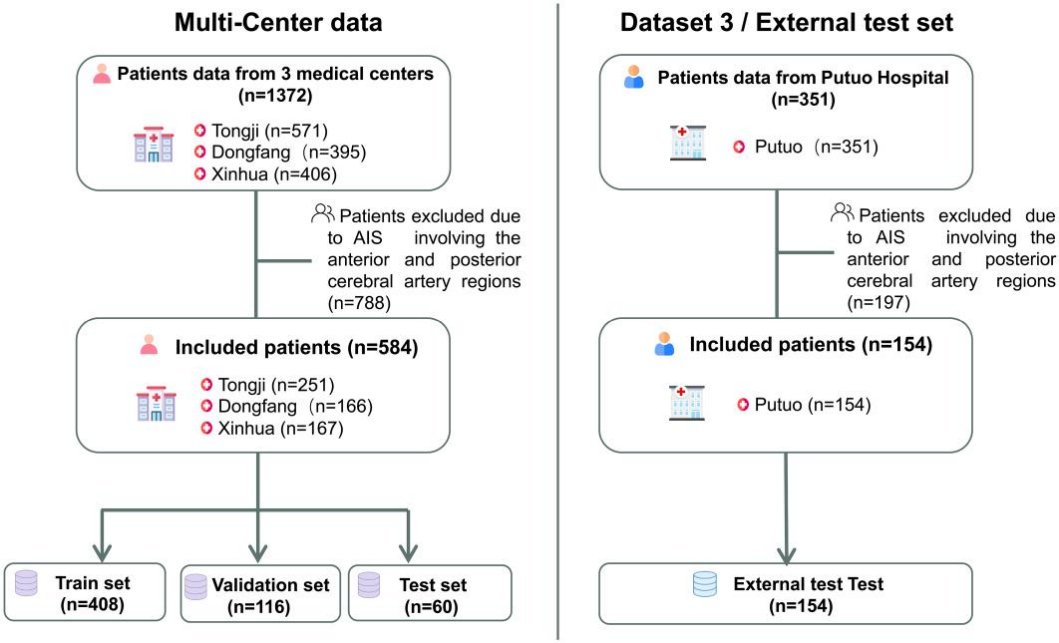
Flowchart of patient selection.

This multi-centre retrospective study was conducted in accordance with the Declaration of Helsinki and was approved by the Ethics Review Committee of Tongji Hospital, Shanghai (Approval No. K-2020 021). The requirement for written informed consent was waived due to the retrospective nature of the study, which posed minimal risk to participants and involved no intervention or alteration of standard clinical care. We pooled individual patient-level data from patients with MCA-AIS admitted to Tongji Hospital affiliated to Tongji University (571 cases), Xinhua Hospital affiliated to the School of Medicine of Shanghai Jiaotong University (406 cases), East Hospital affiliated to Tongji University (395 cases) and Putuo Hospital affiliated to Shanghai Traditional Chinese Medicine University (351 cases) between January 2018 and December 2021. The admission criteria were as follows: (i) patients who had brain MRI (including DWI images) examination within 24 h after symptom onset; (ii) patients initially diagnosed with MCA-AIS who were admitted to the hospital for treatment; (iii) patients who underwent DWI imaging for depicting lesions with a maximum diameter of >1.5 cm; (iv) patients with good quality images without any severe artifacts. The exclusion criteria were as follows: (i) MCA-AIS concurrently involving the anterior cerebral artery and/or posterior cerebral artery territories; (ii) patients who have received endovascular treatment before MRI examination; (iii) patients with lacunar infarcts; (iv) patients with poor quality images. A total of 1372 AIS patients were included in the multi-centre set (Tongji, Xinhua and Dongfang), and 788 patients were excluded due to anterior cerebral artery cerebral AIS (*n* = 172), posterior cerebral AIS (*n* = 142), anterior and posterior AIS (*n* = 102), lacunar AIS (*n* = 356) and image artifacts (*n* = 16). In the external test set (Putuo), 351 AIS patients were included, and 197 were excluded for similar reasons. Ultimately, 584 and 154 cases met the inclusion criteria within the multi-centre set and the external test set, respectively. The multi-centre set was then subdivided into train (*n* = 408), validation (*n* = 116) and internal (*n* = 60) test sets. A flowchart detailing patient inclusion is presented Fig. 2. Among the 608 patients with National Institute of Health stroke scale (NIHSS) score on admission, 390 were classified as mild (NIHSS: 0–7), 158 as moderate (NIHSS: 8–16) and 60 as severe (NIHSS: 17–42).

### MRI-DWI images

The MRI-DWI images were obtained using four different MRI scanners. The acquisition parameters were as follows: (i) Philips Ingenia 3.0T: TR = 2584 ms, TE = 96.7 ms, slice thickness 6 mm, slice spacing 7 mm, field of view 23 cm × 23 cm, matrix 256 × 256, excitation times 2, echo gap 0.75 ms, b value 1000 s/mm^2^; (ii) Siemens Verio 3.0T: TR = 4600 ms, TE = 89 ms, slice thickness 5 mm, scanning without spacing, field of view 24 cm × 24 cm, matrix 256 × 256, echo gap 0.75 ms, b value 1000 s/mm^2^; (ii) uMR 1.5T: TR = 5400 ms, TE = 94 ms, slice thickness 5 mm, layer spacing 6 mm, field of view 23 cm × 23 cm, echo gap 0.75 ms, b value 1000 s/mm^2^ and (iv) GE SIGNA EXCITE 1.5T: TR = 6000 ms, TE = 81.1 ms, slice thickness 7 mm, slice spacing 8 mm, field of view 23 cm × 23 cm, matrix 256 × 256, excitation times 2, echo gap 0.75 ms, b value 1200 s/mm^2^.

### Image pre-processing and registration

The MRI-DWI images were anonymized and converted to NII format. Skull stripping was performed using FSL software (version 6.0.6.5), followed by resampling to achieve uniform spacing with a standard T1-weighted brain template. The publicly accessible T1-weighted average brain imaging served as the template (http://www.bmap.ucla.edu/portfolio/atlases/ICBM_Template/), and a collaborative effort by three senior neuroradiologists culminated in the delineation of the corresponding ASPECTS areas template. The registration process was as follows: first, we aligned the sample orientation with the template, followed by four consecutive registration rounds. The first two rounds consisted of rigid body transformations, while the last two rounds involved affine transformations.

### Image segmentation and DWI-ASPECTS by raters

Three junior neuroradiologists manually outlined the ischaemic lesions on MRI-DWI images with ITK-SNAP software (version 3.8.0, http://www.itksnap.org). The segmentation results were reviewed and refined by three senior neuroradiologists and serve as the gold standard for training the DL model. DWI-ASPECTS scoring was independently completed both on the original DWI images (Raters) and on the DWI images registered with the ASPECTS template (Raters_template). Three junior neuroradiologists evaluated the DWI-ASPECTS, and three senior neuroradiologists reviewed the results. In accordance with the standard ASPECTS scoring criteria,^16^ a point was deducted for lesions in the nucleus area (caudate/internal capsule/lentiform/insular ribbon), and another point was deducted for lesions covering ≥1/3 of the corresponding area in M1–M6.

### 3D U-net segmentation model construction

We used a convolutional neural network model called 3D U-Net based on the PyTorch framework to accomplish the automatic segmentation of cerebral infarction on MRI-DWI images.^17^ This network used 3 × 3 × 3 convolutional kernels for feature extraction, with 2–4 convolutional layers set according to task complexity. The encoder used 2 × 2 × 2 max pooling layers for down-sampling and introduced BatchNorm to accelerate convergence. The decoder upsampled through transposed convolution or bilinear interpolation and employed skip connections to fuse features across different scales.

**Figure 3:**
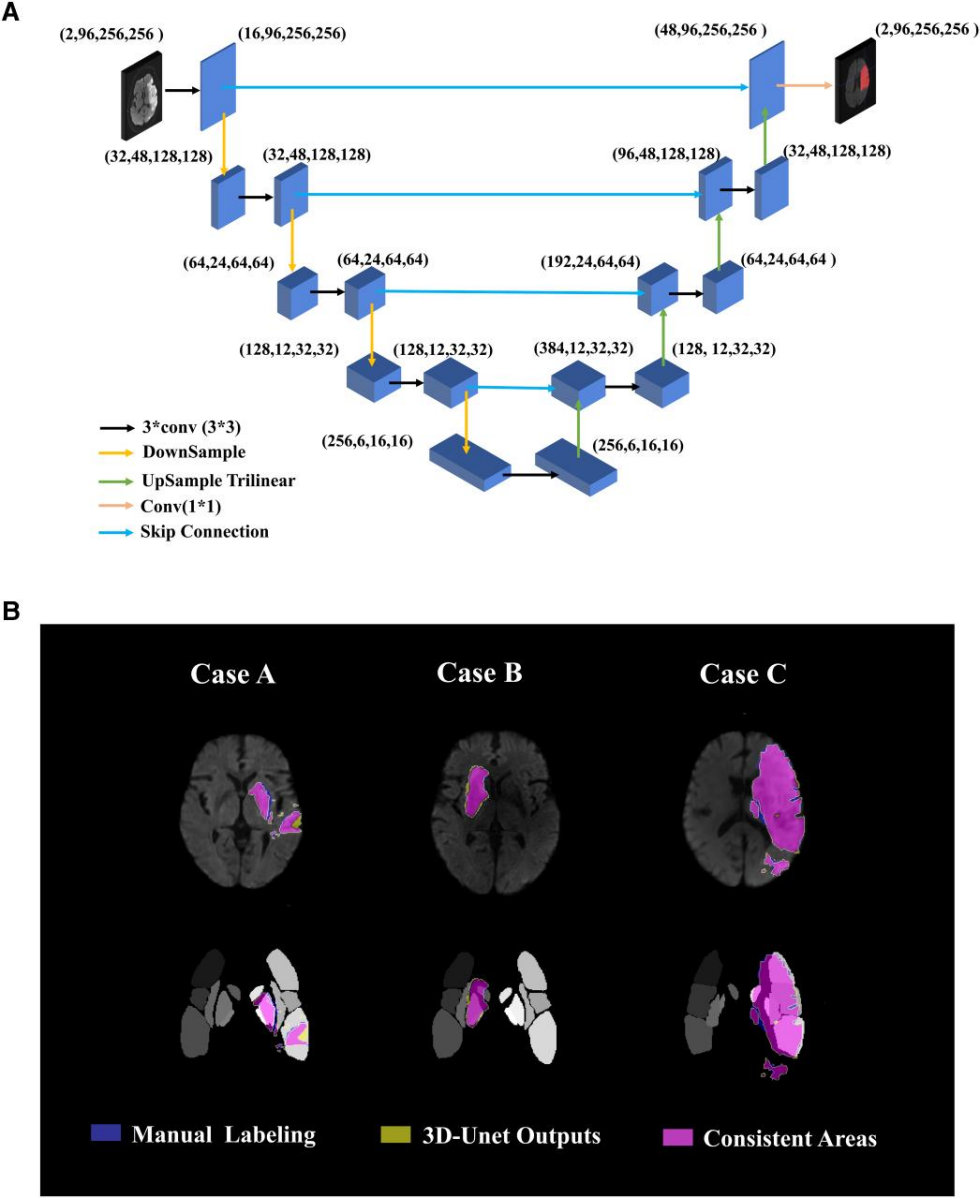
(**A**) The architecture of the 3D-Unet in this study. (**B**) Comparison between automated and manual segmentation. The results are shown for three cases of MCA-AIS from the training set. Segmentation results on DWI images (Upper row); Segmentation results registered with the ASPECTS template (Bottom row). Blue represents manual labelling results, yellow, the 3D-Unet output results and purple, the consistent areas. 3D-Unet = Three-Dimensional U-Net, ASPECTS = the Alberta Stroke Programme Early CT Score.

The optimizer used adaptive Adam, with an initial learning rate set at 1e−2, 100 training epochs and a batch size of 32 (Fig. 3A). We matched the segmentation results post-processing with the ASPECTS template to calculate the ASPECTS score of the samples. Ischaemic core volumes were also quantified, and the model underwent rigorous training, validation and independent performance evaluation on both internal and external test sets to ensure generalizability.

### Statistical analysis

Mann-Whitney U test and chi-square test were used for evaluating significant differences in the variables (such as age and NIHSS score) across the train set, validation set, internal test set and external test set. The Dice coefficient was used to evaluate the performance of DL-based automatic segmentation. The Spearman correlation coefficient (r) was employed to assess the consistency between the lesion volume derived from the DL model and the gold standard volume measured by neuroradiologists. For DWI-ASPECTS, correlation and agreement were measured by the Spearman r and the intraclass correlation coefficient (ICC). Frequency histograms and receiver operating characteristic (ROC) curves were drawn to explain stroke severity. A *P*-value lower than 0.05 was considered to be statistically significant. Statistical analyses, segmentation task, as well as training and validation of the predictive model were programmed using Python (version 3.6).

## Results

### Basic characteristics

Table 1 exhibited the baseline characteristics of included patients in the training, validation, internal and external test sets. As depicted in Table 1, most basic variables of the patients showed no statistical differences (*P* > 0.05) across the training, internal and external test sets, such as general conditions (gender and age), medical history (hypertension and diabetes) and neurological score scales (NIHSS).

**Table 1:** Basic patient information

	Train (*n* = 408)	Validation (*n* = 116)	Internal (*n* = 60)	External (*n* = 154)	*P*-value for Train versus Internal	*P*-value for Train versus External	*P*-value for Internal versus External
Basic characteristics							
Age (Median, IQR)	71 (63, 83)	68 (60, 81)	71 (62, 81)	71 (63, 82)	0.971	0.791	0.900
Male (Percentile: %)	234 (57.3%)	76 (65.5%)	37 (61.6%)	84 (54.5%)	0.527	0.549	0.345
Neurological score scale (Median, IQR)							
NIHSS on admission	5 (3, 10)	5 (3, 9)	5 (3, 7)	5 (2, 10)	0.958	0.828	0.889
Location(Left: Percentile: %)	185 (45.3%)	54 (46.5%)	29 (48.3%)	90 (58.4%)	0.359	0.420	0.181
Neuroimaging score scale (Median, IQR)							
DWI-ASPECTS	8 (7, 9)	8 (7, 9)	8 (7, 9)	8 (8, 9)	0.721	0.088	0.780
History (Percentile: %)							
Alcohol	88 (21.5%)	32 (27.5%)	13 (21.6%)	27 (17.5%)	0.986	0.290	0.486
Smoking	139 (34.0%)	47 (40.5%)	17 (28.3%)	49 (31.8%)	0.379	0.614	0.620
Coronary atherosclerosis	93 (22.7%)	25 (21.5%)	11 (24.5%)	30 (19.4%)	0.438	0.397	0.848
Atrial fibrillation	75 (18.3%)	19 (16.3%)	14 (18.3%)	18 (11.6%)	0.362	0.057	0.032*
Hypertension	302 (74.0%)	80 (68.9%)	43 (71.6%)	113 (73.3%)	0.699	0.877	0.800
Stroke	148 (36.2%)	52 (44.8%)	16 (26.6%)	46 (26.1%)	0.145	0.154	0.114
Diabetes	152 (37.2%)	35 (30.1%)	15 (25.0%)	60 (38.9%)	0.110	0.489	0.230

IQR = interquartile range; NIHSS = National Institute of Health stroke scale. For continuous variables, the MannWhitney U test was used for two independent samples. For categorical variables, if every category had fewer than 10 samples, the Fisher exact test was used; otherwise, chi-square test was used. *P* < 0.05 was considered to indicate a statistically significant difference.

### Segmentation performance of 3D-UNet

The 3D-Unet yielded highly consistent segmentation results across all the train, internal and external test sets. When compared to manual labelling (Fig. 3B), the Dice coefficient was 0.858 and the Spearman r between DL-derived volumes and the manually segmented volumes was 0.982 (95%CI: 0.978, 0.986) in the train set. In the internal and external test sets, the DL model also achieved a Dice coefficient of as high as 0.827 and 0.801, respectively. The high quality of automatic segmentation enabled highly accurate subsequent lesion volume measurement, with Spearman r of 0.985 (0.975, 0.991) and 0.988 (0.983, 0.991) in the internal and external test sets.

### Comparisons of manual ASPECTS versus ASPECTS (Automated) by DL model

In the train cohort (Table 2 and Supplementary Fig. 1), a strong positive correlation was observed between automated ASPECTS scores generated by the DL model (Automated) and manual scores assessed by raters (Raters) (ICC = 0.840; Spearman’s r = 0.759). When neuroradiologists performed scoring on template-registered DWI images (Raters_template), the correlation between Raters and Raters_template (ICC = 0.803; Spearman’s r = 0.733) was comparable to that of Raters versus Automated. Notably, the correlation between Automated and Raters_template significantly improved (ICC = 0.922; Spearman’s rho = 0.914), demonstrating enhanced consistency when utilizing templates for scoring.

**Table 2:** Comparisons of ASPECTS (Raters) and ASPECTS (Raters_template) versus ASPECTS (Automated)

Comparisons	Spearman (r)	*P* value	ICC (95% CI)	*P* value
Train (*n* = 408)				
Raters versus Automated	0.759	<0.001	0.840(0.806, 0.868)	<0.001
Raters_template versus Automated	0.914	<0.001	0.922(0.906, 0.936)	<0.001
Raters versus Raters_template	0.733	<0.001	0.803(0.725, 0.811)	<0.001
Internal (*n* = 60)				
Raters versus Automated	0.729	<0.001	0.829(0.729, 0.894)	<0.001
Raters_template versus Automated	0.895	<0.001	0.913(0.853, 0.948)	<0.001
Raters versus Raters_template	0.731	<0.001	0.828(0.728, 0.894)	<0.001
External (*n* = 154)				
Raters versus Automated	0.694	<0.001	0.798(0.733, 0.849)	<0.001
Raters_template versus Automated	0.903	<0.001	0.920(0.892, 0.942)	<0.001
Raters versus Raters_template	0.712	<0.001	0.800(0.735,0.851)	<0.001

ICC = the intraclass correlation coefficient.

In both internal and external test sets (Table 2 and Supplementary Figs 2, 3), the Spearman’s r values were 0.729 and 0.694 for Raters versus Automated, 0.731 and 0.712 for Raters versus Raters_template and 0.895 and 0.903 for Raters_template versus Automated, respectively. Similarly, excellent agreement was observed based on ICC values, which were 0.829 and 0.798 for Raters versus Automated, 0.828 and 0.800 for Raters versus Raters_template and 0.913 and 0.920 for Raters_template versus Automated in the internal and external test sets, respectively. These results highlight the high consistency between Automated and Raters, as well as the superior consistency between Automated and Raters_template, further validating the accuracy and reliability of the DL model.

### Explaining acute stroke severity

**Figure 4:**
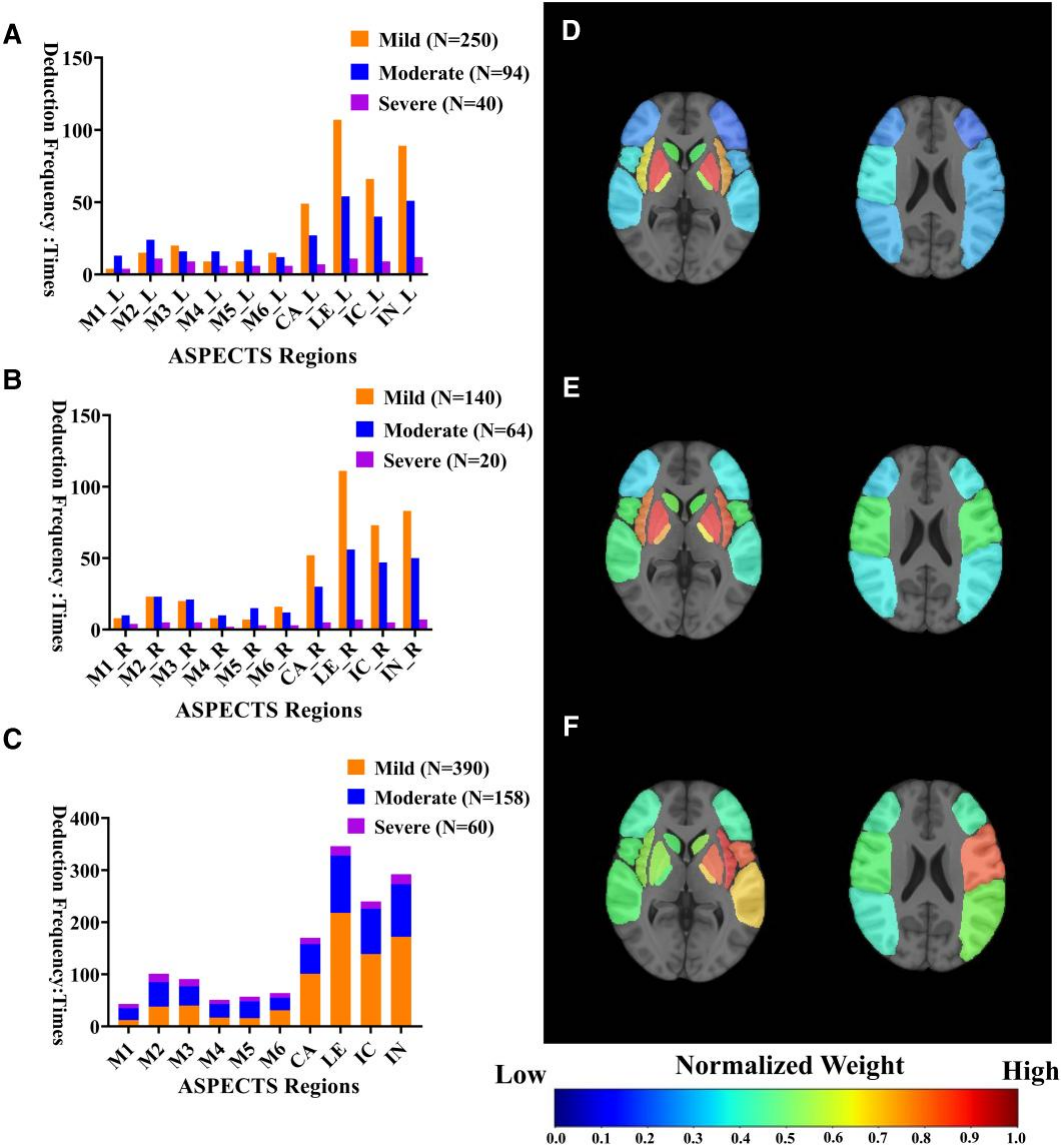
**The cumulative histogram of deduction frequency in ASPECTS regions for MCA-AIS with different degrees: (A)** patients with left MCA-AIS, **(B)** patients with right MCA-AIS and **(C)** all the included patients. The normalized deduction frequency chart of ASPECTS regions in MCA-AIS with different degrees, the colour bar scale from 0 to 1 represents the deduction frequency from low to high: **(D)** Mild, **(E)** moderate and **(F)** severe. *N* = Numbers of patients, ASPECTS = the Alberta Stroke Programme Early CT Score, CA = Caudate, LE = Lentiform_nucleus, IC = Internal_capsule and IN = Insular_ribbon.

Successively, we aimed to interpret acute stroke severity with the DWI-ASPECTS deduction outputs by our DL model. The deduction frequency histogram of the 10 ASPECTS regions in MCA-AIS patients exhibited substantial similarity across the left and right hemispheres in explaining acute stroke severity (Fig. 4A–C). However, notable statistical differences were observed in the deduction frequency of specific ASPECTS regions in the MCA-AIS with different degrees (Supplementary Table 1). Five ASPECTS areas (M1, M2, M3, M5 and M6) significantly contributed to explaining stroke severity, exhibiting a statistical disparity in the deduction frequency among mild, moderate and severe MCA-AIS (*P* < 0.001). Moreover, three ASPECTS regions [lentiform_nucleus (LE), caudate (CA) and Insular_ribbon (IN)] demonstrated a statistical difference between mild and moderate–severe groups. MCA-AIS involving multiple ASPECTS regions often clinically presented as moderate–severe stroke, regardless of the left and right hemispheres, while mild strokes were relatively observed in nuclear regions [LE, CA) and IN].

A normalized deduction frequency cumulative chart based on ASPECTS regions was constructed to illustrate the differences. With respect to individual ASPECTS regions, the largest weights were assigned to the bilateral-hemispheric CA areas for mild MCA-AIS (Fig. 4D). A lesion pattern involving the bilateral-hemispheric M2 and M5 areas, combined with the right-hemispheric M3 and left-hemispheric insular ribbon regions, was more relevant in patients with moderate MCA-AIS (Fig. 4E). Although the severe group exhibited a significantly higher frequency for how often the cortical regions (M1–M6) were affected, particularly the left-hemispheric insular ribbon, M2 and M5 areas. These territories correspond to the anterior central gyrus, posterior central gyrus and temporal gyrus, which are primarily responsible for sensorimotor functions, language and perception (Fig. 4F).

**Figure 5:**
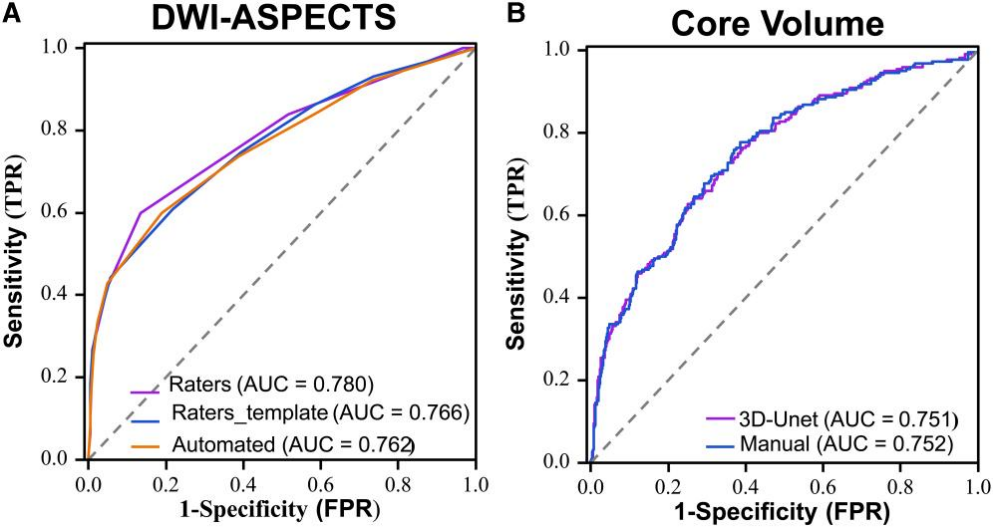
**Receiver operating characteristic (ROC) curves of DWI-ASPECTS and core volume for distinguishing the mild group and moderate-severe group of the MCA-AIS. (A)** DWI-ASPECTS performance. The optimal threshold value on ROC curve analysis was 6. **(B)** Core volume performance. The optimal threshold value on ROC curve analysis was 27.86 mL. DWI = Diffusion-weighted imaging, ASPECTS = Alberta Stroke Programme Early CT Score, TPR = true positive rate and FPR = False positive rate.

According to these results, we used DWI-ASPECTS and Core Volume in different patterns to distinguish between the mild group and moderate–severe group and to compare their performance. For DWI-ASPECTS assessment (Table 3 and Fig. 5A), the Raters yielded an area under the curve (AUC) of 0.780 (95% CI: 0.741–0.819); the Raters_template achieved an AUC of 0.766 (95% CI: 0.726–0.806); and the Automated followed closely with an AUC of 0.762 (95% CI: 0.720–0.803). In terms of Core Volume estimation (Table 3 and Fig. 5B), manual segmentation (Manual) resulted in an AUC of 0.752 (95% CI: 0.713–0.790), closely matched by the 3D-Unet with an AUC of 0.751 (95% CI: 0.712–0.791). The Delong test revealed that there were no statistically significant differences in the ROC curves among the various methods (*P* > 0.05, Supplementary Table 2). When utilizing the outputs generated by the DL model, the optimal threshold values for distinguishing mild from moderate–severe MCA-AIS, as determined by ROC curve analysis with the Youden index, were an automated DWI-ASPECTS of ≤6 (area under the curve 0.761, *P* < 0.001; sensitivity 0.601 and specificity 0.805) and a core volume of ≥27.86 mL (area under the curve 0.788, *P* < 0.001; sensitivity 0.797, specificity 0.645).

**Table 3:** Performance of DWI-ASPECTS and core volume across various assessment patterns

	AUC	Sensitivity	Specificity	Accuracy
DWI-ASPECTS				
Raters	0.780	0.865	0.599	0.769
Raters_template	0.766	0.782	0.608	0.719
Automated	0.762	0.810	0.599	0.735
Core Volume				
Manual	0.752	0.777	0.614	0.664
3D-Unet	0.751	0.627	0.749	0.712

AUC = area under curve.

## Discussion

This multi-centre study successfully developed and validated a DL model for automated DWI-ASPECTS and ischaemic core volume assessment, and the model enabled comprehensive analysis of stroke severity by integrating anatomical regional involvement and volumetric quantification. The DL-based DWI-ASPECTS assessment demonstrated comparable performance to expert neuroradiologists’ evaluations, while the 3D U-Net-derived ischaemic core volume exhibited excellent agreement with manual segmentation. Cortical regions (M1–M6) showed significant relevance in moderate–severe MCA-AIS, with the left hemispheric insular ribbon, M2 and M5 regions being most frequently involved in the severe group. ROC curve analysis established optimal thresholds of 6 for DWI-ASPECTS and 27.86 mL for core volume to distinguish mild from moderate–severe MCA-AIS.

Recently, DL has been applied to the analysis of stroke imaging, including lesion segmentation, disease classification and prognosis prediction.^18-20^ Although the ASPECTS system has been widely utilized to determine the eligibility criteria of mechanical thrombectomy, the lack of agreement and the variability of ASPECTS among even experienced clinicians have been the main source of its limitation. DWI-ASPECTS, which is based on ASPECTS measurement using DWI instead of CT, has been suggested as an alternative and shown to provide a superior inter-rater agreement. Recent studies have proven automated ASPECTS evaluation to be non-inferior and similar to that of experienced neuroradiologists and superior to that of junior stroke physicians.^7-9^ Luu-Ngoc Do *et al*. developed a recurrent residual convolutional neural network for classification between low (1–6) and high (7–10) DWI-ASPECTS groups with an accuracy of 87.3%, an AUC of 0.941 and F1-score of 0.888.^7^ XiaoQing Cheng *et al*. reported that eDWI-ASPECTS software exhibited a diagnostic performance similar to senior neuroradiologists’ evaluation based on ASPECTS region template, demonstrating a strong positive correlation (Kendall’s t a u – b = 0.848) and excellent agreement (ICC = 0.939).^8^ In the external test set, our model demonstrated a Spearman’s r of 0.903 and an ICC of 0.920 between the Raters_template and the Automated. This performance is comparable to that reported in prior single-centre studies, such as the ICC of 0.939 achieved by commercial software in Cheng *et al*., and surpasses the 87.3% accuracy reported by Do *et al*. for dichotomized ASPECTS classification.^8,11^ It is worth mentioning that this study also underwent standard external validation. In the external set, the Spearman r and ICC between Raters_template and Automated were high at 0.903 and 0.920, respectively, corroborating XiaoQing Cheng’s findings. Another highlight of this study was that we categorized manual scoring into two types: scoring using ASPECTS templates (Raters_template) and scoring based on visual evaluation (Raters). Our analysis revealed that while consistency between Raters and Raters_template, as well as between Raters and Automated, was relatively low, the use of ASPECTS templates significantly improved consistency between Raters_template and Automated. These findings elucidate the underlying causes of ASPECTS scoring variability in prior studies, highlighting the challenges even experienced neuroradiologists face in precisely delineating the boundaries of the ten ASPECTS regions without template guidance. Additionally, visual estimation of whether infarct volume exceeds one-third of the total region volume in M1–M6 areas remains highly subjective and prone to error, further contributing to inter-rater variability. However, our model effectively addressed these limitations by enabling rapid and accurate localization, segmentation and quantification, thereby demonstrating the significant advantages of DL approaches in clinical stroke imaging.

Infarction volume plays a pivotal role in the management of AI patients and has shown to be an important factor in determining the eligibility of reperfusion therapy and predicting clinical outcomes.^21,22^ Previous studies have indicated that the AI methods have increased popularity in DWI lesion segmentation, especially the deep learning approaches.^12-15^ For infarct core segmentation, our model achieved a Dice coefficient of 0.801 in the external test set, which aligns with the range (0.79–0.89) reported in another deep learning-based segmentation study.^13^ However, our study is the first to simultaneously output two important imaging quantitative indicators for AIS assessment based on segmentation: DWI-ASEPCTS score and infarction core volume.

Furthermore, we try to explain the acute stroke severity with different degrees based on the outputs by the DL model, a focus that has not been addressed in previous studies. There was a statistical difference in the deduction frequency for most ASPECTS areas among mild, moderate and severe MCA-AIS. It was found that ischaemic lesions were more extensive in moderate–severe AIS patients, while mild strokes predominantly involved small infarcts in the nucleus regions.^23-25^ Nuclear regional infarcts are typically caused by occlusions of cerebral perforating arteries (e.g. lenticular artery), whereas main middle cerebral artery occlusions often result in multiple cortical and nuclear infarctions. Moderate-severe MCA-AIS, primarily involved the left inferior frontal gyrus and temporal lobe, suggests detrimental effects on language, motor and sensory functions. The involvement of right-hemispheric frontotemporal regions, which impaired the integrity of the domain-general network and resulted in neurological damage, was more pronounced in the moderate–severe patients. A previous study also found that affected left and right subcortical regions were similar in explaining acute stroke severity, whereas left cortical affected regions additionally included inferior frontal, insular and superior temporal gyrus regions, as well as the post-central gyrus, which just supported our results.^26^

The DWI-ASPECTS and Core Volume output by the DL model effectively differentiated between the mild and moderate–severe MCA-AIS, exhibiting diagnostic performance that aligned closely with the gold standard. Our findings also indicated that MCA-AIS patients with DWI-ASPECTS ≤ 6 and Core Volume ≥ 27.86 mL possess a higher risk of experiencing moderate–severe stroke in clinical practice. As we know, DWI-ASPECTS includes overestimations in the penetrating branch territory of the MCA (caudate/internal capsule/lentiform/insular ribbon) because a small volume lesion involving these regions can lead to a low score. In contrast, regions outside the MCA territory and coronal radiation area are not accounted for in ASPECTS, which may lead to large-volume lesions with high ASPECT scores. That’s why the diagnostic efficacy of DWI-ASPECTS and Core Volume could be complementary. The findings from authoritative articles^10,11^ indicated that ASPECTS and Core Volume serve as important imaging markers in the selection of treatment plans for AIS. Contrasting with the time-consuming and laborious process of traditional manual scoring and segmentation methods, the DL model developed in this study can efficiently generate these two key quantitative imaging indicators.

In conclusion, our 3D-Unet model offers a reliable solution for automated DWI-ASPECTS and ischaemic core volume assessment with performance matching that of experienced neuroradiologists. We designed the model for seamless adoption into daily practice, allowing it to function as a standalone platform or integrated directly into the Picture Archiving and Communication System. By analyzing uploaded DWI sequences in seconds, the system produces an immediate quantitative report that highlights involved regions, calculates the ASPECTS score and renders the infarct core in 3D. This capability transforms the model into a vital decision-support tool for acute stroke teams. It streamlines emergency workflows by assisting neurologists and interventionalists with rapid triage, prognosis assessment and evidence-based treatment planning.

### Limitations of the study

First, the test sets included a higher proportion of cases with relatively smaller infarct core areas, necessitating a larger study cohort in future research to broadly generalize the performance of the DL model. Nevertheless, our study benefited from certain strengths compared to the majority of prior research,^7-13^ particularly the multi-centre design and the implementation of a standardized training and validation process. Second, we only used the outputs of the DL model to explain the stroke severity; further research should combine the ASPECTS region-specific information with clinical information to aid in the selection of candidates for reperfusion therapy and accurately analyse the long-term prognosis of stroke. Third, the exclusion of very small infarcts (maximum diameter ≤1.5 cm) reflects the primary clinical objective of our study: to develop an automated tool specifically for the rapid and objective assessment of non-lacunar MCA-AIS. Consequently, the applicability of our proposed model to lacunar or minor infarcts has not yet been established and requires further validation in future studies.

## Supplementary Material

fcag053_Supplementary_Data

## Data Availability

The original code for this study has been archived at https://github.com/ecust-sl/3Dunet/tree/master. The data presented in this article can be obtained from lead contact upon request.
