# Supplementary material for: Automated quantification of the Alberta Stroke Programme Early CT Score (ASPECTS) and infarct core on diffusion-weighted imaging in acute ischaemic stroke: multi-centre validation and severity stratification
Source: Brain Commun. 2026 Feb 24;8(2):fcag053. doi: 10.1093/braincomms/fcag053 (PMC12971011; doi:10.1093/braincomms/fcag053)
Supplement: fcag053_Supplementary_Data [file fcag053_supplementary_data.pdf]

Supplementary Figure 1

N=408

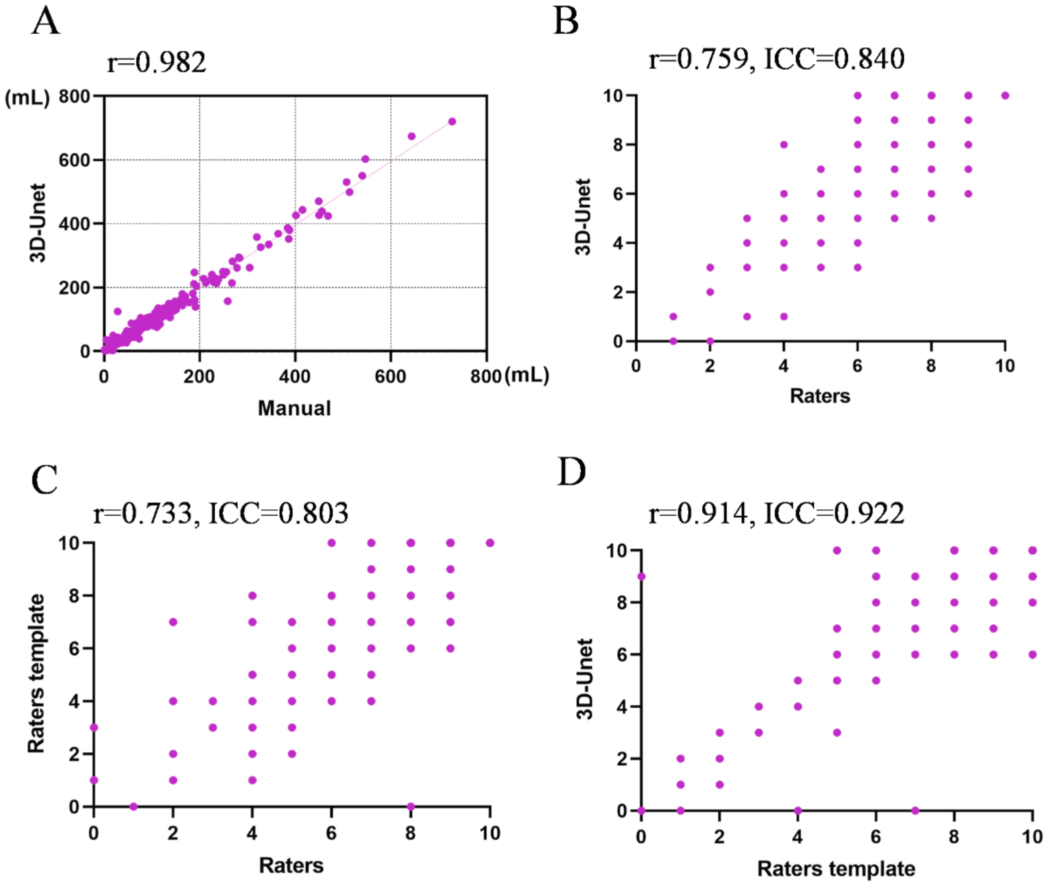

Supplementary Figure 1. Correlation plots： each point represents one or more patients with identical score pairs. The correlation between AI-derived volumes and the manually segmented volumes in train set (A). There were a strong positive correlation and excellent agreement between manual ASPECTS score by naked eyes (Raters), manual ASPECTS score with template (Raters_template) and automatic ASPECTS score by 3D-Unet (Automated) in train set (B, C, D). Spearman correlation (r) was used to assess linear relationships. The intraclass correlation coefficient (ICC) for absolute agreement between the two respective methods (k=2) was applied to evaluate inter-method and inter-rater reliability.

Supplementary Figure 2

N=60

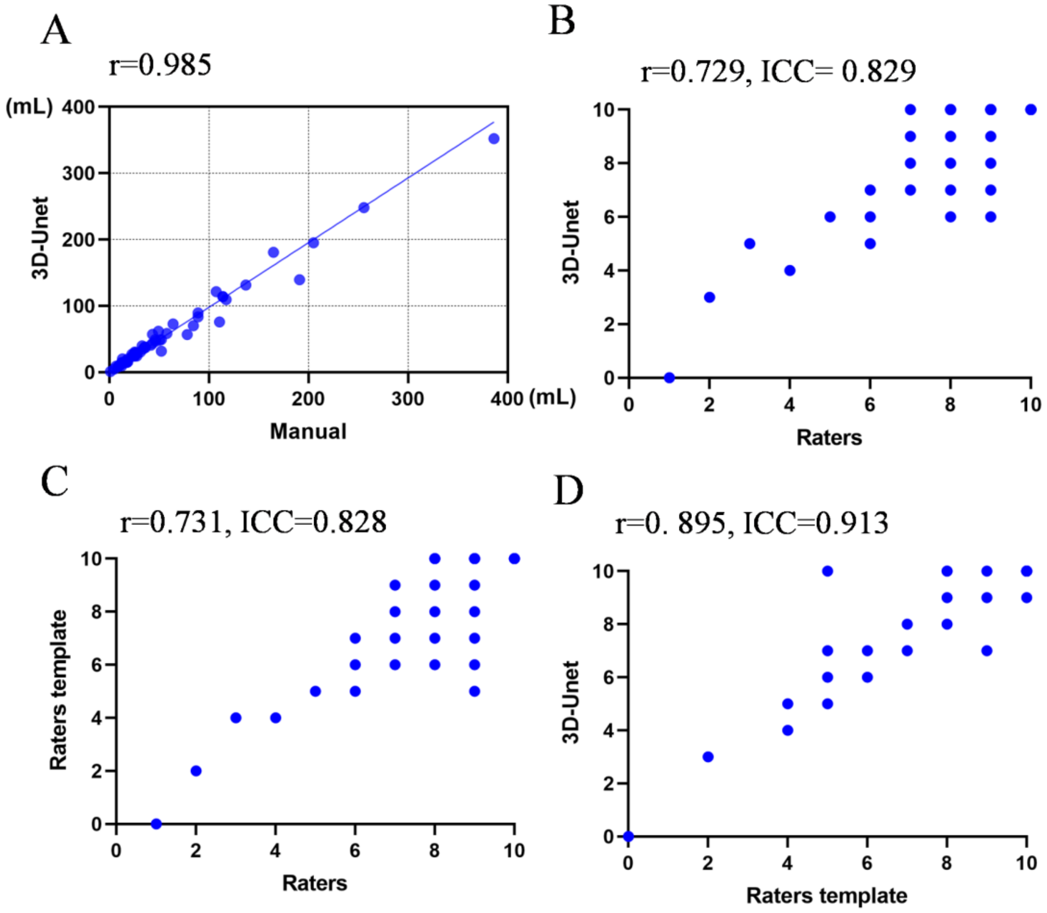

Supplementary Figure 2. Correlation plots: : each point represents one or more patients with identical score pairs. The correlation between AI-derived volumes and the manually segmented volumes in internal test set (A). There were a strong positive correlation and excellent agreement between manual ASPECTS score by naked eyes (Raters), manual ASPECTS score with template (Raters_template) and automatic ASPECTS score by 3D-Unet (Automated) in internal test set (B, C, D). Spearman correlation (r) was used to assess linear relationships. The intraclass correlation coefficient (ICC) for absolute agreement between the two respective methods (k=2) was applied to evaluate inter-method and inter-rater reliability.

Supplementary Figure 3

N=154

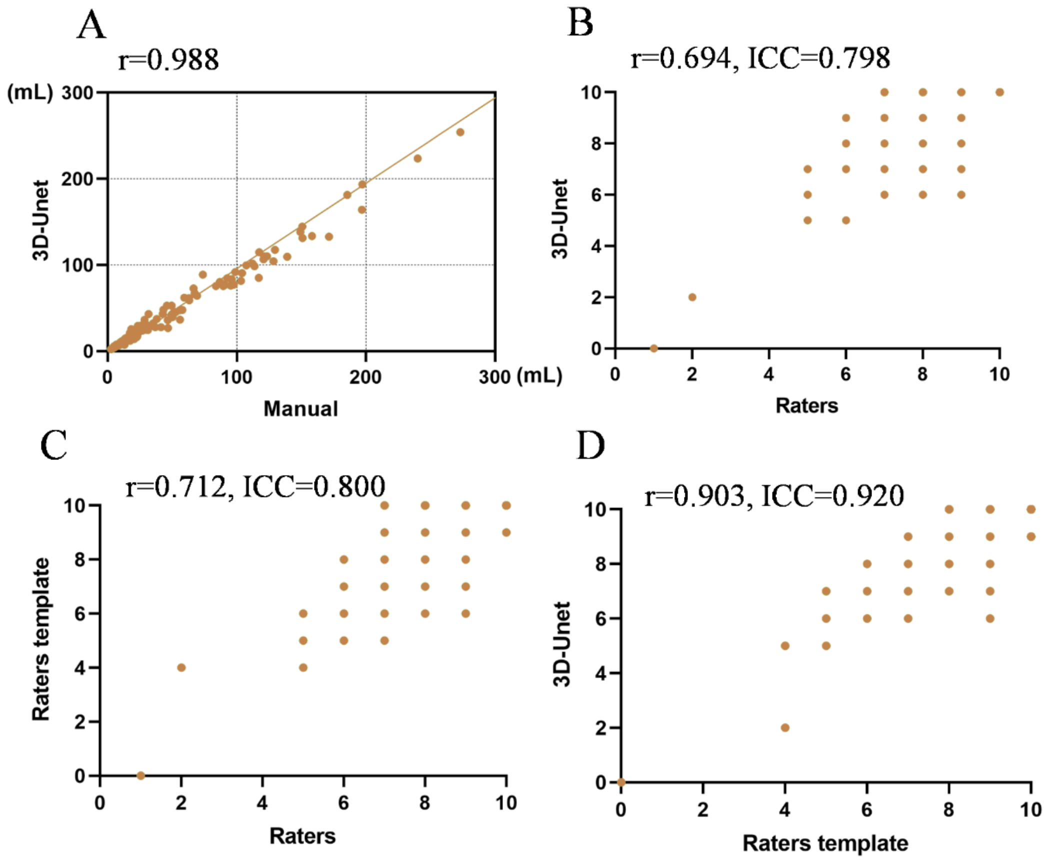

Supplementary Figure 3 Correlation plots: each point represents one or more patients with identical score pairs. The correlation between AI-derived volumes and the manually segmented volumes in external test set (A). There were a strong positive correlation and excellent agreement between manual ASPECTS score by naked eyes (Raters), manual ASPECTS score with template (Raters_template) and automatic ASPECTS score by 3D-Unet (Automated) in external test set (B, C, D). Spearman correlation (r) was used to assess linear relationships. The intraclass correlation coefficient (ICC) for absolute agreement between the two respective methods (k=2) was applied to evaluate inter-method and inter-rater reliability.

Supplementary Table 1 Comparisons of deduction frequency of ASPECTS regions in MCA-AIS with different degrees.

ASPECTS regions	Comparisons	chi-square	p value
**M1**	**A** vs. **B**	29.01	<0.001***
	**B** vs. **C**	6.774	0.009**
	**A** vs. **C**	66.263	<0.001***
**M2**	**A** vs. **B**	48.063	<0.001***
	**B** vs. **C**	15.155	<0.001***
	**A** vs. **C**	106.390	<0.001***
**M3**	**A** vs. **B**	30.223	<0.001***
	**B** vs. **C**	11.101	<0.001***
	**A** vs. **C**	71.795	<0.001***
**M4**	**A** vs. **B**	28.643	<0.001***
	**B** vs. **C**	3.487	0.062
	**A** vs. **C**	47.660	<0.001***
**M5**	**A** vs. **B**	45.696	<0.001***
	**A** vs. **C**	10.446	0.001**
	**B** vs. **C**	100.640	<0.001***
**M6**	**A** vs. **C**	16.732	<0.001***
	**B** vs. **C**	15.209	<0.001***
	**A** vs. **C**	68.426	<0.001***
**CA**	**A** vs. **B**	16.600	<0.001***
	**B** vs. **C**	2.947	0.086
	**A** vs. **C**	93.586	<0.001***
**LE**	**A** vs. **B**	64.017	<0.001***
	**B** vs. **C**	0.778	0.378
	**A** vs. **C**	37.684	<0.001***
**IC**	**A** vs. **B**	3.470	0.062
	**B** vs. **C**	0.275	0.600
	**A** vs. **C**	3.291	0.070
**IN**	**A** vs. **B**	110.210	<0.001***
	**B** vs. **C**	6.127	0.013*
	**A** vs. **C**	93.581	<0.001***

A=Mild group, B=Moderate group, C=Severe group, CA=Caudate, LE=Lentiform_nucleus, IC=Internal_capsule, IN=Insular_ribbon, *indicates significant difference, **indicates highly significant difference, ***indicates extremely significant difference.

Supplementary Table 2 DeLong's Test for ROC Curves of DWI-ASPECTS and Core Volume Across Various Assessment Patterns.

	z statistic	p value
**DWI-ASPECTS**		
**Raters** vs. **Automated**	1.205	0.228
**Raters_template** vs**. Automated**	0.769	0.441
**Raters** vs. **Raters_template**	0.914	0.361
**Core Volume**		
**Manual** vs. **3D-Unet**	0.297	0.766

DWI=Diffuse-Weighted Imaging; ASPECTS=The Alberta Stroke Program Early CT Score.
